# Design and Characterization of a Low-Cost and Efficient Torsional Spring for ES-RSEA

**DOI:** 10.3390/s23073705

**Published:** 2023-04-03

**Authors:** Omar Sabah Al-Dahiree, Raja Ariffin Raja Ghazilla, Mohammad Osman Tokhi, Hwa Jen Yap, Mustabshirha Gul

**Affiliations:** 1 Department of Mechanical Engineering, Faculty of Engineering, University of Malaya, Kuala Lumpur 50603, Malaysia; omarsmech@gmail.com (O.S.A.-D.);; 2 School of Engineering, London South Bank University, London SE1 0AA, UK; 3 Department of Mechanical Engineering, Faculty of Engineering and Technology, Bahauddin Zakariya University, Multan 60000, Pakistan

**Keywords:** force sensor, series elastic actuator, SEA, torsion spring, lumbar support, wearable robot exoskeleton, lifting task

## Abstract

The design of torsional springs for series elastic actuators (SEAs) is challenging, especially when balancing good stiffness characteristics and efficient torque robustness. This study focuses on the design of a lightweight, low-cost, and compact torsional spring for use in the energy storage-rotary series elastic actuator (ES-RSEA) of a lumbar support exoskeleton. The exoskeleton is used as an assistive device to prevent lower back injuries. The torsion spring was designed following design for manufacturability (DFM) principles, focusing on minimal space and weight. The design process involved determining the potential topology and optimizing the selected topology parameters through the finite element method (FEM) to reduce equivalent stress. The prototype was made using a waterjet cutting process with a low-cost material (AISI-4140-alloy) and tested using a custom-made test rig. The results showed that the torsion spring had a linear torque-displacement relationship with 99% linearity, and the deviation between FEM simulation and experimental measurements was less than 2%. The torsion spring has a maximum torque capacity of 45.7 Nm and a 440 Nm/rad stiffness. The proposed torsion spring is a promising option for lumbar support exoskeletons and similar applications requiring low stiffness, low weight-to-torque ratio, and cost-effectiveness.

## 1. Introduction

### 1.1. Background

With heavy lifting and manual material handling (MMH), lower back pain (LBP) is a significant factor contributing to work-related musculoskeletal disorders (WMSD). Intense compression pressure on the spine from heavy lifting can cause degeneration, fractures, or chronic damage to the lumbar spine [1]. By using off-body mechanical devices such as forklifts and trolleys to transport heavy loads, workers may reduce the risk of musculoskeletal injuries during MMH. Although lifting aids can alleviate the burden of heavy loads on labourers [2], they are often not used if the loads are within human capacity [3]. To address this issue, exoskeleton technology is being hailed as a promising new approach to support the human body and reduce the risk of musculoskeletal injuries. This technology involves wearing a device to support and protect workers’ health [4]. An exoskeleton with active actuators can also enhance human strength for load-handling tasks.

In traditional robotic systems, especially industrial applications, stiff actuators are employed for high-position precision, stability, and high torque bandwidth. However, for physical human–robot interaction (pHRI), such as in rehabilitation, assistive, and service robotics, the actuators must prioritize safety and comfort for human operators. As a result, these types of robotic systems often use compliant actuators to increase adaptability and safety. The compliant actuator allows deviation from the preset position when the external load changes. Rigid actuators, on the other hand, maintain their position regardless of external force. Compliance in the actuation system can increase adaptability and safety [5].

To address the drawbacks of rigid actuators and ensure safe human–robot interaction, series elastic actuators (SEAs) have been widely used in robotics. In a SEA, there is an elastic element connected to the mechanical energy source [6]. This architecture allows for improved force (or torque) control and reduces the influence of inertia and friction [7]. Using SEAs with closed-loop controls and active force sensors reduces the impact of inertia and friction. Hooke’s Law can be used to calculate the force on the output load based on the deflection of the compliant part. A feedback force controller adjusts the motor’s current to compensate for discrepancies between actual and intended forces [8]. SEAs can also reduce the cost of robotic systems by using electrical current in the joints to estimate forces and movements instead of expensive sensors [9].

SEAs have proven successful in several robotic applications, including wearable robots such as bipedal, quadruple, and dual-arm robots [10]. Because SEAs are naturally compliant for safe human–robot interaction, they are well-suited for assistive exoskeleton applications. Two studies in the literature have used SEAs for lumbar support exoskeletons at the hip joints [11,12]. One exoskeleton uses four SEA units with clutches to assist with hip abduction and adduction, as well as hip flexion and extension [11]. The other exoskeleton uses a SEA-based wire-driven mechanism that reduces muscle activity by 33.0% and 41.6% in semi-squatting postures [12]. However, wearable robots would be more effective if they were more compact and lightweight than current SEA designs, which are bulky and heavy. To address this, a novel energy storage-rotary series elastic actuator (ES-RSEA) has been developed, as detailed in our previous publication [13,14], to assist with squat lifting tasks. The compact and modular design of the ES-RSEA leverages the biomechanics of the human squat strategy to utilize the kinetic energy of limbs for semi-squat lifting tasks.

The main challenge for SEA technology is the dependence of SEA performance on spring stiffness. A balance must be struck between compliance and torque transmission. High force control and compliance come from low-stiffness springs, while high torque transmission efficiency is achieved with high-stiffness springs, but at the cost of reduced compliance and control. High torque estimation accuracy requires high deflection linearity with torque relation. Choosing the right spring stiffness based on application biomechanics ensures high compliance and maximum force. Designing a torsional spring that is low in stiffness and robust in torque is challenging, especially in wearable robotics, where weight and dimensions must be minimized.

### 1.2. Torsional Springs for a Rotary Series Elastic Actuator: State of the Art

The design of torsional springs in series elastic actuators (SEAs) plays a crucial role in determining the system’s overall performance. As the deflection of a torsional spring in a SEA can be used to measure the applied torque, it can also be considered a type of force sensor. The torsional spring in a SEA acts as the interface between the actuator and the load, transmitting torque while also measuring the force being applied. Therefore, the torsional spring’s design must be carefully considered to ensure accurate torque measurement and efficient force transmission.

Much effort has been devoted to creating unique modules customized for particular applications in SEA development. Since the purposes of SEA are so diverse, there are currently no standard solutions for elastic components, and various designs of elastic components have been implemented with different specifications. Torsional springs incorporated into the transmission drive or custom torsional springs attached directly to the load are often used in compliant systems [7]. The single-element elasticities are best suited to developing a lightweight and portable method that meets the spring needs of a single application; Table 1 shows the main specifications for the current designs of torsional springs.

Two different types of custom-designed torsional springs have been used in the literature. The spring blades are positioned equally along the output shaft and bent sinuously. Because of this design, the SEA’s space waste is dramatically decreased, and the deflection angle is significantly enhanced. In contrast to the first form of changing the spring leaf into a personalized pattern, the second type is the spiral spring design, which is regarded as a standard shape based on the traditional mathematical design of the Archimedes spiral.

**Figure 1:**
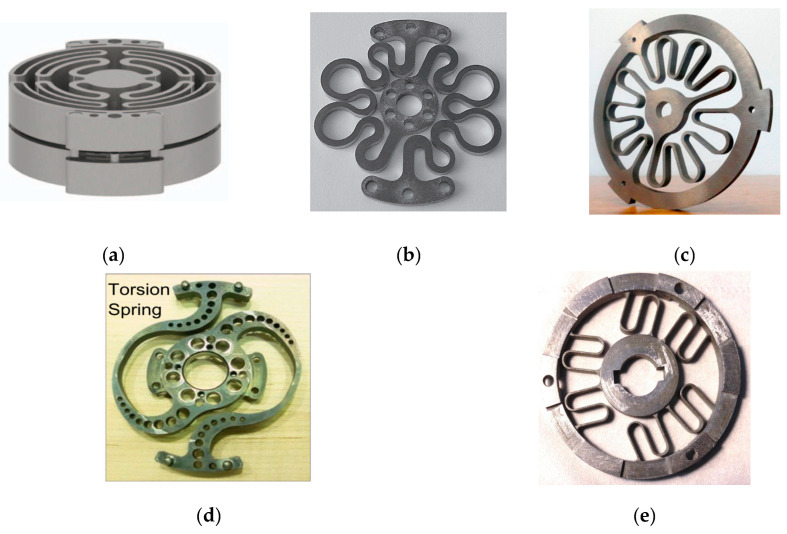
Patterns of compliant elements for rotary series elastic actuators. (**a**) two identical custom torsion springs [16]. (**b**) compact torsion spring, adapted with permission from Ref. [17]. 2023 Copyright Omar Al-Dahiree. (**c**) customized torsion spring, adapted with permission from Ref. [18]. 2023 Copyright Omar Al-Dahiree. (**d**) torsion spring for Valkyrie’s series elastic actuator, adapted with permission from Ref. [19]. 2023 Copyright Omar Al-Dahiree. (**e**) torsional elastic module [22,23].

Most springs were designed using finite element analysis (FEA), as reported in references [7,10,15,16,17,18,19,20,21,22,23,24,25,26], and some of these springs are depicted in Figure 1. In reference [15], a custom-made spiral spring is presented, which was developed based on prior research work depicted in [10]. The double spiral design of the spring mitigates undesirable radial forces that would otherwise act on the centre of the spring during torque strain. The spring was designed using fundamental beam bending equations and ANSYS Workbench FEA simulation data. Another type of spiral-shaped torsion spring is a custom spiral-shaped [27]. This spiral spring is made from a steel strip and fitted with the Kurmet biped robot, whereas the other varieties of spiral springs are made from a steel plate using electrical discharge processing. In these earlier studies, a spring design configuration was chosen based on the application requirements and then modelled in an FEA methodology. The FEA study is iterated and improved with incremental design parameter changes until a solution meets performance standards while remaining within packaging constraints. However, other researchers used double planner torsional springs in a series of connections to suit their application requirements, as reported in [7]. Moreover, as illustrated in Figure 1b, work [17] develops a compact torsion spring for a series elastic actuator for an active orthosis. The spring’s form was determined using FEM-based geometry optimization to reduce the maximum equivalent stress. 

**Table 1:** Comparisons of the main features of existing torsion spring design.

Spring	Diameter (mm)	Thickness (mm)	Weight (g)	Torque (Nm)	Torsion Stiffness (Nm/rad)	Angular Deflection (Degree)	Material	Weight/Torque Ratio	Application
A [15]	75	15	235	100	219	26	Maraging steel 350	2.35	Gait rehabilitation training (stroke survivors)
B [10]	60	10	Unknown	50	150	Unknown	Maraging steel 350	Unknown	Upper limb rehabilitation
C [16]	90	11	370	60	250	13.7	Maraging steel 300	6.17	Gait assistance and human augmentation
D [7]	85	3	61.5	7.7	98	4.4	Maraging steel C-300	7.98	Gait assistance and human augmentation
E [17]	60	10	96	15	172(3)	Unknown	56Si7 and 55Cr3 steel	6.4	Stroke rehabilitation device for motor impairments
F [18]	125	6	292	15	84	10	Chromium–Vanadium steel (AISI 6150)	19.46	Stroke rehabilitation, gait training
K [25,26]	90	5.6	121.7	70	400	10	50CrVA	1.7	Gait assistance and human augmentation
L [22,23]	60	5	60	4	60.2	4.98	60Si2Mn	15	Gait assistance exoskeleton

All the initial designs have been developed and used in various exoskeletons or orthosis applications. The torsional spring was designed for clutchable SEA, which is used for lifting tasks in the hip exoskeleton and has a peak torque of 60 Nm and a spring stiffness of 800 Nm/rad [11]. This spring has a disc-shaped construction to reduce the size and weight of the torsion spring. This spring’s additional design features were unavailable. Then, alternative spring designs created the clutchable SEA for the hip exoskeleton, again for assistive gait [25,26]. It demonstrates that the disc-shaped spring can transfer a maximum torque of 70 Nm with a 400 Nm/rad stiffness (N). Table 1 outlines the essential features of torsion springs, excluding those for which data is unavailable.

In general, finding a torsional spring component that satisfies the requirements for low stiffness, compactness, and lightweight design while still capable of handling high-peak torques can be challenging. The existing designs are mainly based on researcher perspectives rather than optimized for performance. There is a lack of standard design methodology for torsional springs, making the development of a novel topological torsion spring that meets the specific design requirements of energy storage-rotary series elastic actuator (ES-RSEA) for lifting task exoskeleton applications highly desirable. This torsion spring design must be lightweight, compact, and withstand high torques while maintaining low inherent stiffness. Existing torsional springs in the market often lack these features, making investigating a new design imperative for the success of the ES-RSEA or any other SEA for lifting task applications.

### 1.3. Design Objectives of the Proposed Torsional Spring

This article aims to design a compact, lightweight, and cost-effective torsional spring with excellent torsion-compliant properties for use in the energy storage-rotary series elastic actuator (ES-RSEA) of a lumbar support exoskeleton. The torsional spring must handle high assistive torques while ensuring compliance to support the human operator during lifting tasks and meet the actuator’s low weight and size requirements. The methodology considers factors such as design for manufacturability (DFM), material selection, and cost-effectiveness. A low-cost material, such as AISI 4140 alloy steel, has been selected for its yield strength of up to 1400 MPa after heat treatment and a safety factor of 1.65. This material selection balances cost-effectiveness with performance requirements.

## 2. Design Considerations

### 2.1. Design for Manufacturing (DFM) Approach

Design for Manufacturing (DFM) principles were employed in the torsion spring design to ensure compatibility with the ES-RSEA actuator. DFM is a design approach that aims to simplify, optimize, and refine product designs for easy manufacturing and to achieve a better final product at a lower cost. The optimization process in DFM offers several optimal solutions for the manufacturing process and selects the best solution among them [28,29]. DFM considers the manufacturing difficulties at the product development stage, which reduces overall manufacturing cost and improves product reliability and quality, and reduces complexity without sacrificing standards [30]. 

The DFM approach used for the torsion spring in this study was based on ease of manufacture and assembly and consistency with the manufacturing capability to meet the product’s criteria [31]. The goal was to manufacture the torsion spring using existing technologies available at the research institute or in the local market. The DFM guidelines identified design features that are challenging to manufacture using machining processes and thus need to be avoided, such as insufficient or inaccurate designs, the excessive number of holes with widely divergent diameters, nonstandard hole diameters, tight tolerances, and sharp corners [32].

The manufacturing method for the spring was chosen based on several criteria, including manufacturing cost, machine availability, and cutting quality. The machining process for the torsion spring was carried out using a water jet cutting machine, which offers efficient, safe, and highly accurate cutting [33]. The limitations of the water jet machine were considered in the design process, such as the minimum permissible thickness of the flexible spring segment (set at 2 mm) and the width of the material plate (set at 8 mm). Additionally, the nozzle size of the waterjet cutting stream was considered when calculating the space for designing the flexible segment between two rings. The outside ring was designed with the highest permissible diameter of 85 mm to provide enough space for compliance design.

Material selection was an essential part of the design process. Different materials, such as maraging steels and titanium alloys, have been used in the literature, but these high-cost materials were not considered in this work due to cost-effectiveness concerns. Chromium-molybdenum alloy steel AISI 4140 was chosen for its low cost, wide availability, and high fatigue and torsional strength, yielding 1400 MPA after heat treatment at 315 °C. Low-alloy steel material has good machinability and high ductility and can be produced using traditional methods in the annealed state.

### 2.2. Mechanical Design Considerations

**Figure 2:**
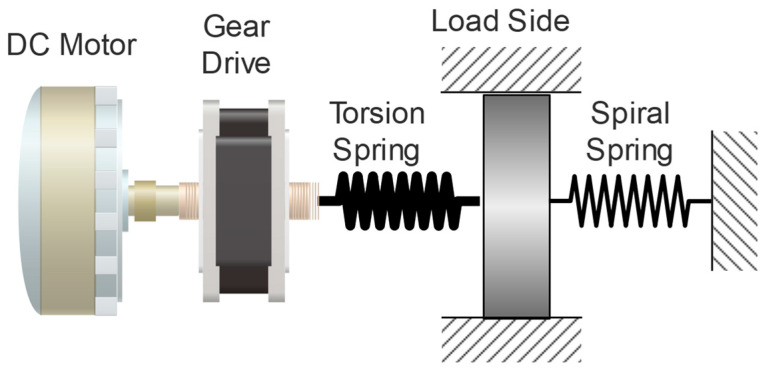
ES-RSEA configuration.

The ES-RSEA actuator was designed for use in a lumbar support exoskeleton to provide torque assistance during the ascent and descent phases of lifting tasks [13]. The rotary series elastic actuator (RSEA) and the energy storage device (ES) work together to achieve this goal. The RSEA provides mechanical compliance and a high torque-weight ratio at the interface between the user and the exoskeleton, reducing the risk of injury and protecting both the human body and the exoskeleton structure. The design and topology of the actuator affect the shape and properties of the torsional spring, which is the main component of the RSEA. The ES-RSEA configuration is shown in Figure 2. The ES-RSEA has a nominal torque of 45.7 Nm and a spring stiffness of 450 Nm/rad, chosen for its optimal performance in terms of accuracy, bandwidth, compliance, and torque transmission efficiency [13]. Further details on the stiffness selection are discussed in the next section.

**Figure 3:**
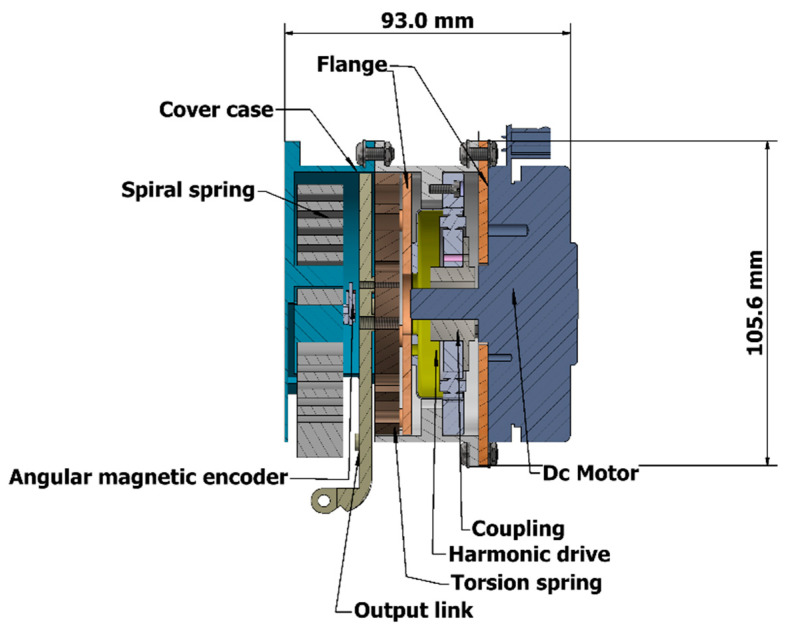
Cross-section of ES-RSEA.

The ES-RSEA is designed to be lightweight and compact, using a planar torsional spring with a maximum weight of 150 g. The elastic element (torsional spring), located between the harmonic drive and load side, connects the external ring of the spring to the harmonic drive gear through a thin flange and the inner ring (20 mm diameter) directly to the load link [13]. The torsional spring can be designed using a simple model of two rings connected by a flexible segment for the desired compliance. The shape of the flexible segment can be optimized through the mass reduction factor and FEA design. The ES-RSEA actuator has a compact design with an axial length of 93 mm. Figure 3 illustrates the cross-section of the ES-RSEA actuator [13]. According to the characteristics, topology, and cutting technology accuracy, the specific design parameters of the torsional spring are summarized in Table 2.

### 2.3. Selection of Spring Stiffness

The spring stiffness selection plays a crucial role in the performance and characteristics of series elastic actuators (SEAs). The accuracy of measuring an elastic element’s displacement and applied torque in a SEA depends on the suitable spring stiffness [7]. This is because the spring deflection is used to measure the applied torque, and a more compliant spring allows for less torque signal quantization, which improves torque accuracy [7]. It is also important to have high linearity in the relationship between the applied torque and angular displacement to provide accurate torque estimation and effective control performance [7].

Furthermore, the output torque of the actuator relies on the deflection and spring stiffness. An overly stiff spring may cause discomfort to the human operator and result in inaccurate control of torque fidelity. Hence, the suitable spring stiffness must be chosen carefully as a trade-off between a large torque bandwidth and low compliance. The torque range accuracy and motor control performance should also be considered when designing the spring characteristics [34]. 

The spring stiffness influences the torque measurement resolution as the exerted torque is computed from the spring displacement [7]. High deflection linearity with torque relation should be ensured to achieve high torque estimation accuracy and torque transmission performance [35]. Selecting the proper spring stiffness for a given application will produce a more energy-efficient system [34]. To sustain high compliance throughout the task cycle while maintaining the ability to deliver maximum force, choosing the correct SEA springs based on specific application biomechanics is necessary. This process balances controller bandwidth and high torque resolution [17].

The relationship between a torsional spring’s torque resolution and maximum angular deflection depends on its design features such as stiffness, wire diameter, and material properties. The maximum angular deflection represents the maximum twisting or rotation the spring can undergo before it reaches its limit, while torque resolution measures the smallest amount of torque to produce a noticeable change in angular deflection. Typically, a torsional spring with a higher stiffness has a higher torque resolution but a lower maximum angular deflection, and vice versa. However, the relationship can vary based on the torsional spring’s specific design, and the optimal design may require a trade-off between these two factors based on the application’s specific requirements and constraints [16,18,36].

The suitable spring stiffness value of series elastic actuators (SEAs) in recent studies has been found to range from 100 to 1600 Nm/rad based on literature research [37,38,39]. Most SEAs use high-stiffness springs to provide high-force transmission efficiency, which can compromise compliance, back drivability, and force control performance [8]. Theoretical studies and simulations have shown the applicability of these values as a trade-off between the numerous design requirements of SEAs.

The appropriate value of the physical elasticity for the hip joint when designing a wearable robot for lifting activities is challenging to determine. This is because assessing the lower limb joints’ viscoelastic characteristics has revealed varied findings for each examined joint in biomechanics research and wearable robotics literature [7]. Currently, most SEAs developed for hip exoskeletons have a stiffness of approximately 800 Nm/rad [40,41,42,43,44,45,46]. Therefore, 200–800 Nm/rad stiffness was selected as the design target for the lower limb exoskeleton for lifting tasks.

The proposed torsion spring for ES-RSEA has a stiffness of 450 Nm/rad, which was chosen based on the requirements of the lumbar support exoskeleton and the desired level of compliance in human–robot interaction. While the stiffness value may be considered higher than the lowest value, it is the minimum value required to achieve the desired level of performance. The proposed torsion spring is a promising option for lumbar support exoskeletons and similar applications requiring low stiffness, low weight-to-torque ratio, and cost-effectiveness. The designed torsion spring for the ES-RSEA has a 450 Nm/rad stiffness, a bearing torque of 45.7 Nm, and a maximum angular deflection of 5.8 degrees.

## 3. Topology Design of Torsion Spring

**Figure 4:**
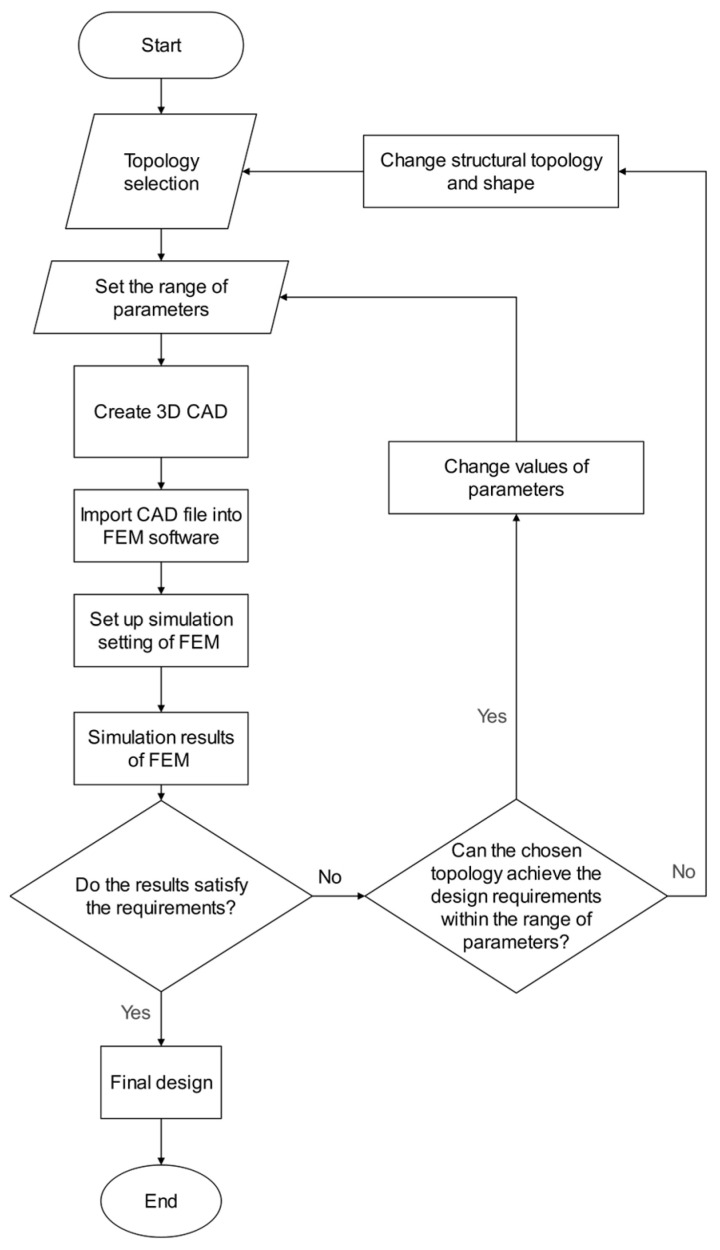
Flowchart of the spring design process.

The design of the torsion spring involves two main stages. The first step is determining the topologies with specific parameters that meet the design and DFM requirements. The second step is to optimize the spring design parameters based on the chosen topology using FEM-based geometry optimization to minimize the equivalent stress and achieve the desired spring stiffness while ensuring a lightweight design. The flowchart in Figure 4 summarizes the design process of the torsion spring.

Initially, a set of topologies is selected, each defined by a set of parameters. A finite element analysis (FEA) program is then used to evaluate the design requirements of each topology. If the design requirements are not met, the following options are available:

- Adjust the parameters within the acceptable range for the selected topology [47].
- If the parameter search space for the specified topology has been fully explored, change the structural topology and repeat the process (the topology is discarded after 20 iterations if the design requirements are still unsatisfied).

The design optimization phase concludes when the design criteria are satisfied (i.e., the spring stiffness is approximately 450 Nm/rad), and the chosen topology is deemed a good design. This design methodology ensures that at least one feasible design meets the design objectives outlined in Table 2.

### 3.1. Selection of the Suitable Topology

The proposed torsion spring consists of two main parts: the inner coil and the outer coil. The flexible element between the two coils provides the desired compliance and stiffness of the spring and induces elastic deformation when the inner and outer coils rotate relative to each other. The flexible element also acts as a bridge to transfer torque between the coils and reduce impact. Its shape and geometry can be developed based on mass reduction and FEA-based optimization designs. The outer ring’s maximum exterior diameter and inner ring’s maximum interior diameter are 85 mm and 20 mm, respectively, as per the human hip joints and ES-RSEA structure.

Topology optimization was conducted to determine a compliant design for the flexible elements that meet the application requirements. It was concluded that the elastic element should have a more extended force transmission path, as this leads to reduced equivalent stresses, and the desired stiffness can be achieved with larger cross-sections.

**Figure 5:**
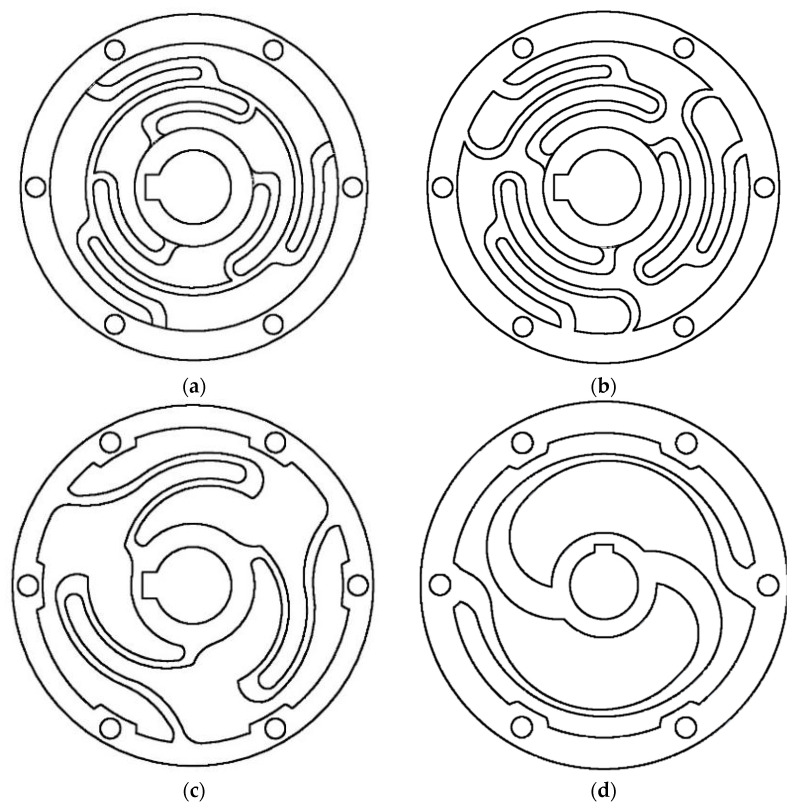
Elastic element module topologies considered: (**a**) Topology A; (**b**) Topology B; (**c**) Topology C; and (**d**) Topology D.

Four elastic elements with symmetric structural topologies were investigated and are shown in Figure 5. Each element’s topology was modified until it reached the optimum topology of Element D. The number and arrangement of the flexible components are important, but their dimensional characteristics determine how the elements are morphologically implemented. The first three topologies have symmetric structures with three elastic elements in radial replication, while the fourth topology has two symmetrical elastic elements. Elements A, B, C, and D were examined during the geometry identification process. The elastic element was modelled as follows:

- Parameter range values for each topology were determined and applied.
- The parts were modelled in Autodesk Inventor (2020) using parametric CAD.
- The CAD file was imported into ANSYS Workbench (2022 R2, Canonsburg, PE, USA) for FEM-based geometry optimization.
- Design requirements-based simulations were conducted to achieve the desired spring characteristics.

The stiffness and linearity of the element were tested using iterative FEM in ANSYS through static performance analysis, and the optimal structure was found through optimization and selection procedures. Topologies A, B, and C showed unpromising results for achieving the spring characteristics with design and DFM considerations. Element A was too rigid and could not provide the desired spring deflection, despite having the lowest equivalent stress among all the topologies. Element B had improved output deflection but did not reach the desired stiffness and had higher equivalent stress than Element A. Both elements A and B were considered complicated and costly to manufacture due to their many arched lamellae, edges, and curves. Element C had a higher equivalent stress than Element A but lower than Element B. Its deflection reached higher than both previous designs. However, due to its sharply arched lamellae, the maximum equivalent stress exceeded the maximum allowable stress for the chosen material at the root (stress concentration). The deflection of this element almost achieved the desired stiffness, but it did not fulfil the DFM recommendations.

Elastic element thickness was reduced in certain areas due to adapting the geometry of element C to selected spring properties, resulting in disadvantages regarding the calculated equivalent stresses, shape accuracy, and design complexity. Therefore, element C failed to fulfil the DFM considerations to meet the required spring characteristics. This was also true for elements A and B.

The design of a flexible element needs to be compliant enough to achieve the desired spring deflection, but if the material is rigid and not highly ductile, it may result in high stress under the applied torque. Element D was designed with a sufficient torque transmission path length to reduce the generated stress and provide the capability to deform the element to achieve the desired spring stiffness and deflection. It has a smooth corrugation for the arched lamellae that reduces radial deformation and distributes the resulting stress evenly throughout the circumference during twists. Element D can provide the desired stiffness and deflection without reaching high equivalent stresses, making it a viable solution in terms of design considerations and requirements. It offers potential for improvement in optimizing parameters to attain the desired spring characteristics while still considering maximum equivalent stress and mass reduction. As a result, topology D was chosen as the best design solution for the torsion spring.

### 3.2. Schematic Design

**Figure 6:**
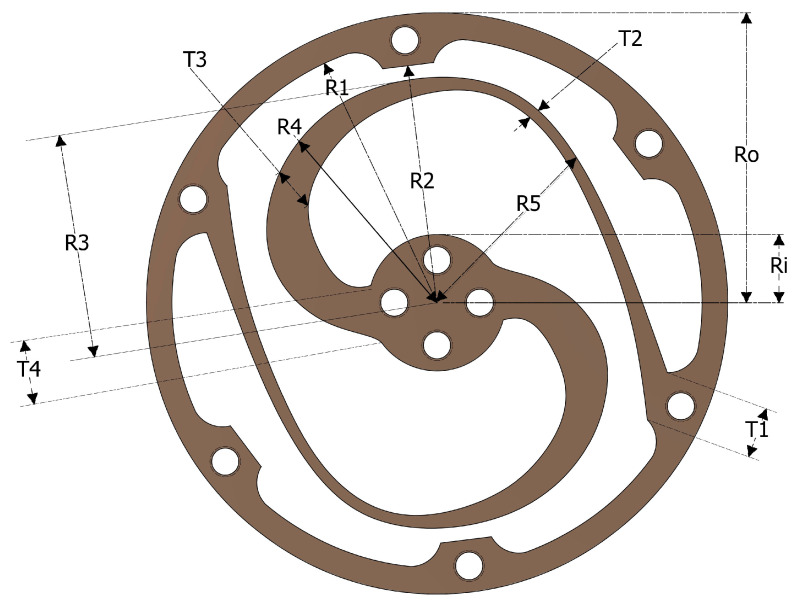
Morphology of the chosen topology.

This section describes the schematic design of the torsion spring. The design process starts with creating a concept for the search range of parameters, which are used to develop the schematic design of the torsion spring. A specific shape for the flexible element D, which consists of two worm-shaped lamellae separated by a distance of π from each other, is specified and illustrated in Figure 5 and Figure 6. The elastic elements are arched lamellae distributed in a wide loop between the inner and outer circles, acting as worm-shaped lamellae.

Constant parameters such as the radius of the inner (Ri) and outer rings (Ro), the inner radius of the outer ring (R1 and R2), and the distance (T1) between the rings are defined and depend on the selected hole size (M4.5) and the allowable edge distance from the hole. A rule of thumb is to place the bolt hole centre 1.6 times the bolt diameter from the edge of the outer ring to avoid exceeding the tear-out strength.

Considering each replication of the lamellae block, the distances along the path of the lamellae from the centre are defined as R3, R4, and R5. T2, T3, and T4 define the widths of lamellae at different locations along their paths. The thickness of the spring module is labelled with a “t”. Each variable distinctly impacts the spring’s flexibility, stability, and overall strength. Hence, optimising a parameter based on the FEM experimental design methodology is possible. Table 3 summarizes the design parameters, with minimum and maximum allowable bounds and a minimum increment for each parameter, which are adopted into the search space for the optimization process using the finite element method (FEM).

### 3.3. Finite Element Method (FEM) Based Design Approach

In the design of a torsion spring, a finite element method (FEM) based approach was used to determine the stress distribution and deformation of the chosen spring topology. ANSYS Workbench 2022 R2 was used for the linear static structural analysis to ensure the highest equivalent stress was below the material’s yield strength (1400 MPa with a safety factor of 1.65) under the applied torque of 45.7 Nm. The FEM simulation was part of an iterative design optimization process to find the optimal values for the search space parameters (as shown in Table 3) while reducing weight and size but maintaining the required spring characteristics.

The FEM verification was based on four criteria: spring stiffness meeting design requirements, limit deformation angle meeting requirements, spring topology meeting DFM design guidelines, and no failure under the maximum torque with equivalent stress below the material’s yield strength. 

In the analysis, two boundary conditions were defined based on the actual mounting state of the torsion spring: the inner ring of the spring was fixed to the ground through its four drill holes, and the torque was applied to the external surface of the outer ring. The spring stiffness (*K*) was calculated by dividing the applied torque by the angular deformation [18], based on Hooke’s Law [48]:

(1) $$K=\frac{T}{\theta }$$

where *T* is the torque and θ is the angular position.

**Figure 7:**
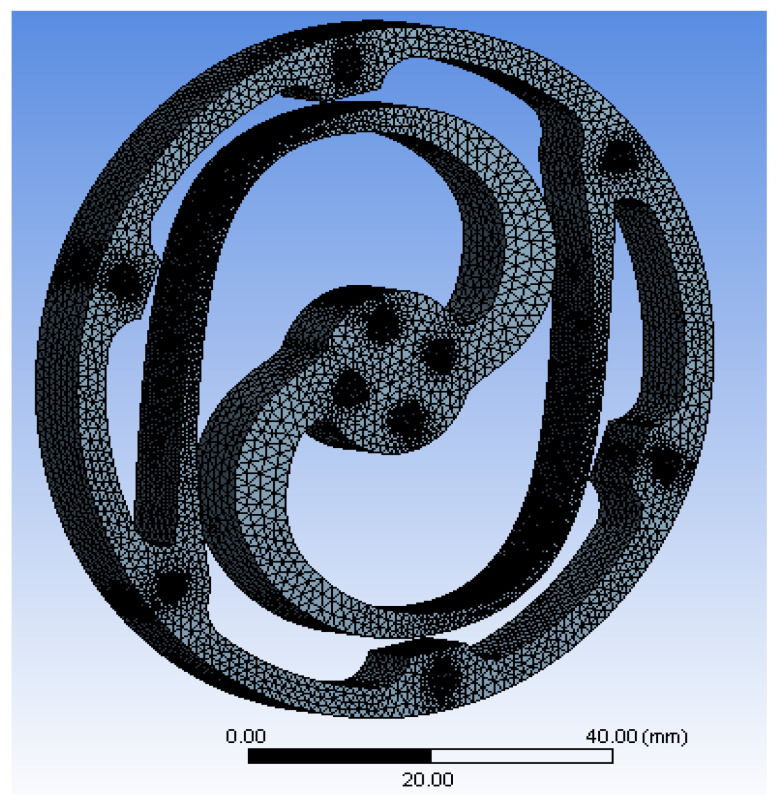
Meshed module.

The mesh utilized in this study is a single-layer swept tetrahedral mesh. The finer mesh surrounds lamellae and holes, which have more curvature and smaller cross-sections, and the coarser mesh surrounds the outer ring, where predicted lower stresses occur. The 3D subdomain was generated by starting with the torsion spring’s surface and sweeping the resulting face mesh through the body, employing advanced function sizes for proximity and curvature and a high smoothing element. Figure 7 illustrates this mesh. Regulations for the structured mesh were made based on a convergence approach to ensure steady mesh generation throughout the computation of many variations.

A mesh analysis of torsion springs was performed before starting the optimization procedure to ensure the precision of the optimization results. The convergence approach ensured a steady mesh generation throughout the optimization process. The element quantity was found to fluctuate from 100,000 to 500,000 nodes. The results of the convergence test showed the appropriate number of nodes ranging from 250,000 to 300,000. Exactly 292,842 nodes were found and used for the actual optimization, with a maximum size of 1.5 mm and a minimum of 0.017587, as shown in Table 4.

### 3.4. Design Verification Using FEM

**Figure 8:**
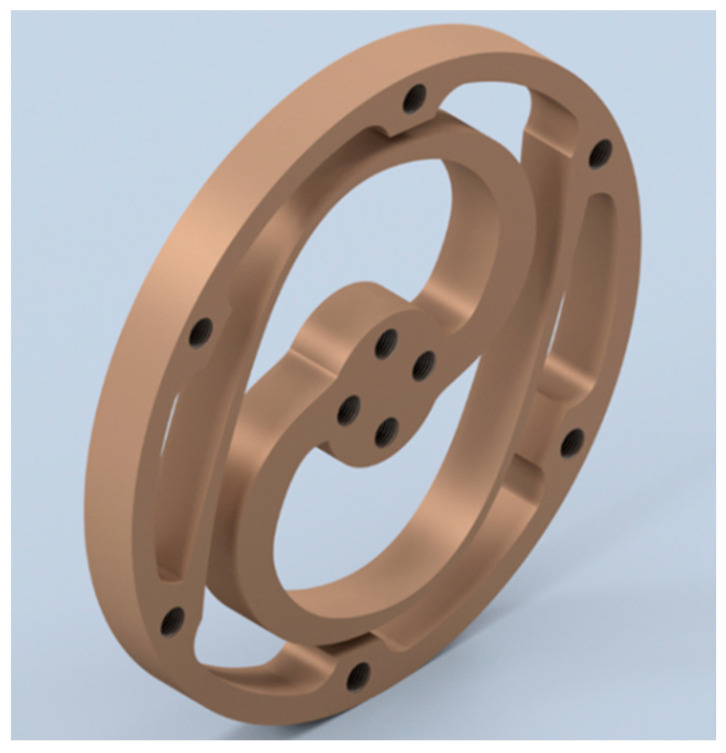
Optimized module for torsion spring.

A finite element method (FEM) analysis was performed to verify and optimise the selected topology structure’s design parameters within the search space shown in Table 3. In Ansys Workbench, multiple finite element analysis simulations were conducted on the optimized structure to evaluate its performance under an applied torque of 45.7 Nm. The results showed that the weakest part of the structure was located at the root of the lamellae segment and near the inner ring, where stress concentration was high. The weakest area was manually strengthened to increase the overall strength and achieve smooth stress distribution. The thin parts of the lamellae segments were accordingly reinforced, resulting in an optimized structure. The optimal geometry parameters are shown in Table 5, and the optimal topology of the spring module is depicted in Figure 8.

**Figure 9:**
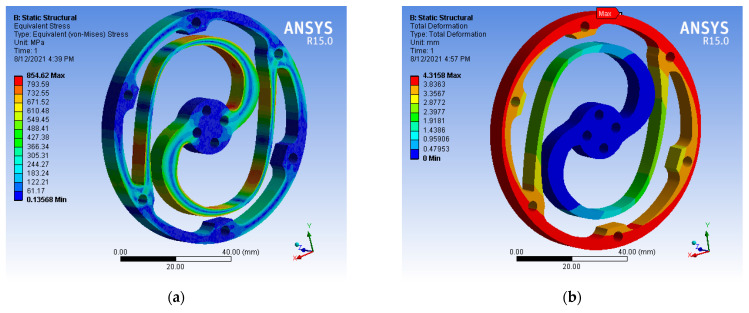
FEM results: (**a**) Module deformation (mm); (**b**) Von Mises stress (MPa).

The thickness of the component was set to an optimal value of 8 mm to maximize material usage and reduce stress while avoiding excessive bulkiness. The results of the FEM simulation for the optimal parameters, including the 1:1 Von Mises stress and the spring module deformation, are shown in Figure 9. The stress distribution in the spring body is within the acceptable range, with a Von Mises stress of 854 MPa and a maximum deformation of 4.3158 mm under the applied torque of 45.7 Nm.

## 4. Experimental Characterization of Torsion Spring

### 4.1. Manufacturing of the Torsion Spring

**Figure 10:**
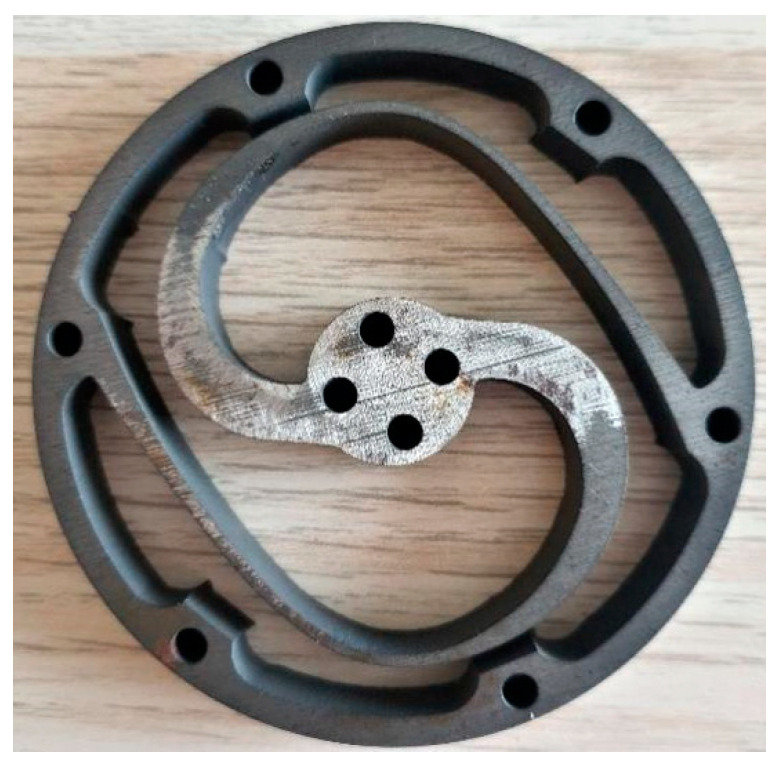
Manufactured torsion spring.

The torsion spring model was optimized using the FEM design technique, as shown in Figure 10. In the early stages of spring design, design for manufacturing (DFM) considerations were taken into account to provide feedback on the spring design and avoid any challenges during the final prototype manufacturing. With DFM, potential issues can be addressed early in the design stage, saving time and money. As a result, the decision to select the appropriate cutting machine for the manufacturing process was made based on DFM considerations and recommendations.

The waterjet cutting machine was selected to manufacture the spring prototype over other methods such as wire electrical discharge machining (WEDM), CNC, and laser cutting because the waterjet cutting process can cut irregular shapes, curved shapes, and internal holes with good edge quality and precision [49]. The waterjet technique is also a cold-cutting process that cuts materials without deformation or heat-affected zones [49]. The available waterjet cutting machine in Malaysia has a cutting accuracy of ±0.1 mm and a nozzle diameter (stream size) of up to 2 mm. The limitations of the used waterjet machine were considered in the early design stage.

Finally, the optimized torsion spring module was manufactured from an 8 mm AISI 4140 alloy steel plate, as shown in Figure 10.

### 4.2. Test Rig Setup

**Figure 11:**
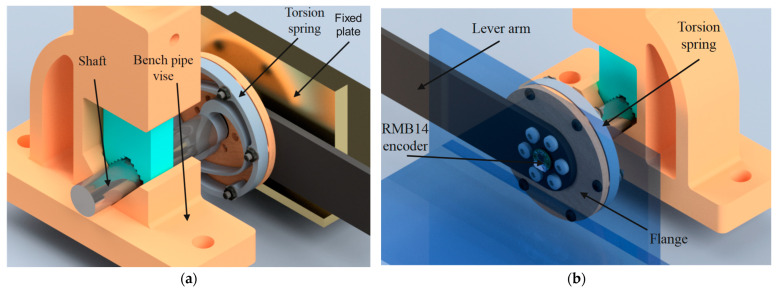
3D rendering of the testbed of the torsion spring: (**a**) Front view and (**b**) Back view.

A test rig was established to evaluate the torsion spring’s elastic properties using the operating procedure of the ES-RSEA actuator. The test rig consisted of a fixed plate, torsion spring, bench pipe vise, steel wire, RMB14 angular magnetic encoder (12-bit absolute, RLS), flange, lever arm, and shaft. The components of the test rig were selected for their low cost, efficiency, and ease of assembly. A 3D CAD rendering of the test rig with the torsion spring is shown in Figure 11.

Four bolts were used to secure the torsion spring’s inner diameter to the steel shaft. The steel shaft was then clamped in place using the pipe jaws of a bench vise, as illustrated in Figure 11. The exterior diameter of the spring was attached to a movable flange on the other side using six bolts. Torque was applied to the exterior diameter of the spring through a lever arm, which was attached to the central axis of the flange. A known mass could be added to the integrated steel wire attached to the lever arm 300 mm away from the spring to produce the estimated torque.

The RMB14 angular magnetic encoder (12-bit absolute, RLS) was selected to track the angular deflection of the torsion spring during the experiment. The lowest measurement increment was 0.0879 degrees, based on the encoder’s resolution (12 Bits). This resolution was sufficient for detecting the deflection angle for stiffness testing, as the applied torque did not fluctuate dynamically. The encoder magnet was positioned on the circular side of the lever arm, parallel to the spring axis, and installed on the fixed plate. 

The load was applied to the moving lever arm, which had a length of 300 mm, through a 1.2 mm diameter 304 stainless steel wire rope. The mass of the load was increased in increments of 1.47 Nm from 0.5 kg to 15.5 kg, corresponding to a range of applied torques from 1.47 Nm to 45.6 Nm. The resulting torque was transmitted to the outer ring of the torsional spring, while the inner ring was mounted to the frame, with a diameter of 20 mm. The actual angular deflection was measured using an RMB14 angular magnetic encoder, which was 12-bit absolute and provided by RLS.

## 5. Results and Discussion

### 5.1. Stiffness Characteristics 

**Figure 12:**
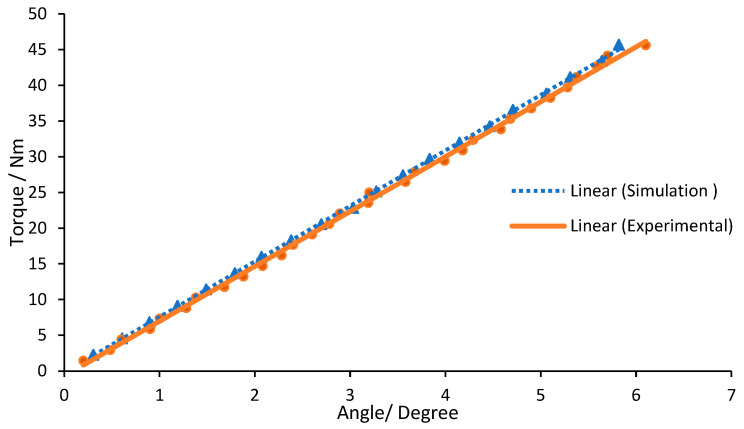
Characteristic of spring stiffness.

This section explains the findings from the simulations and experiments performed on the torsion spring. The torsion spring’s characteristics were accurately predicted using the FEM calculation model, as shown by the simulation results. The maximum vertical displacement of the outer ring was measured to be 4.3158 mm at the rotation arc deformation value, corresponding to a maximum angular rotation of 0.10155 rad under a peak torque of 45.7 Nm. A total of 20 simulations were performed to analyze the linearity of the torque-displacement relationship, applying torque from 2.285 Nm to 45.7 Nm with a step of 2.285 Nm. The resulting values for each simulation are reported with a dashed line in Figure 12. The results show linear characteristics with a torsion stiffness of 445.19 Nm/rad and a high linear regression $R^{2}$ value of 0.9997, comparing with theoretical stiffness $k_{th.}$ of 450 Nm/rad. The spring stiffness matches its design requirement with an error of 1.07%, corresponding to an error between the theoretical value and the simulated value, calculated as $\left(k_{sim.}-k_{th.}\right)/k_{th.}$.

The torsion spring prototypes were tested experimentally. The torque versus angular rotation characteristics of the simulation and experimental torsion spring modules are shown in Figure 12. Five tests were carried out at each torque level, allowing the spring element to return to its initial position following each measurement. The resulting average values are depicted in Figure 12. After linear regression for the data collected, the experimental results show a linear relationship between torque and angular rotation with $R^{2}$ value of 0.9987. The experimental result revealed that the measured spring stiffness $k_{exp.}$ of 440 Nm/rad is roughly 1.166% less than the stiffness $k_{sim.}$ calculated from the FEM simulations of 445.19 Nm/rad, computed as $\left(k_{exp.}-k_{sim.}\right)/k_{sim.}$

The results of the torque-displacement relationship demonstrate that the characteristic stiffness response is remarkably linear within the examined torque range, reflecting over 99% linearity and no significant backlash. The hysteresis was difficult to determine, but the spring deformation was stable and symmetric, as the curve plot illustrates in the figure. The difference between the experimental and simulated results was less than 2%, which is considered a trivial difference and may be attributed to good manufacturing accuracy, accurate FEM-based design optimization, and matching material properties between the prototype and CAD module.

### 5.2. Overall Spring Characteristics 

The proposed torsion spring weighs 140 g, has a maximum angular deflection of 5.8 degrees, an applied torque of 45.7 Nm, and a compact design with a thickness of 8 mm and an outer diameter of 85 mm. The experimental stiffness of the spring was measured at 440 Nm/rad, and the weight-to-torque ratio was found to be 3.

**Figure 13:**
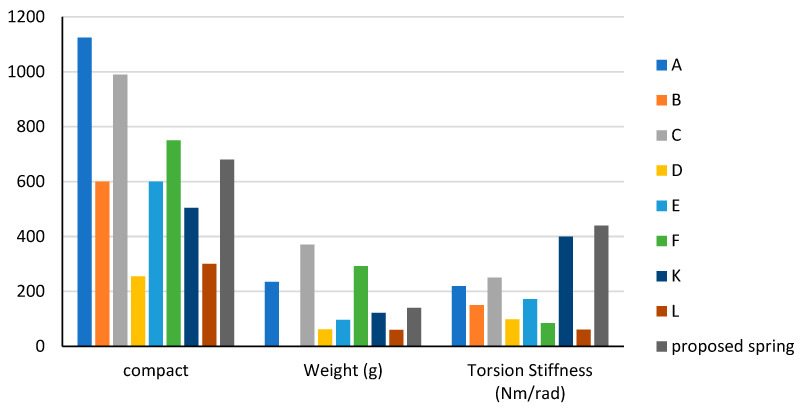
Spring characteristics of compactness, weight, and torsion stiffness. (A) [15]. (B) [10]. (C) [16]. (D) [7]. (E) [17]. (F) [18]. (K) [25,26]. (L) [22,23].

**Figure 14:**
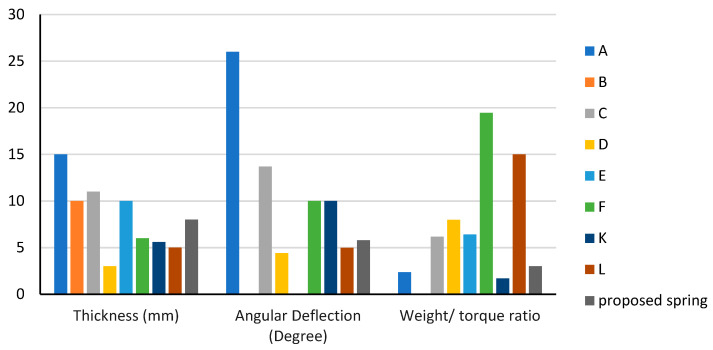
Spring characteristics of thickness, angular deflection, and weight-to-torque ratio. (A) [15]. (B) [10]. (C) [16]. (D) [7]. (E) [17]. (F) [18]. (K) [25,26]. (L) [22,23].

The proposed spring was compared to other existing spring models listed in Table 1 (A, B, C, E, F, K, and L) based on five key characteristics: material cost, compactness, weight-to-torque ratio, stiffness, and maximum angular deflection. The comparison results are shown in Figure 13 and Figure 14 in bar charts.

The comparison results with other spring models in the literature showed that the proposed spring has the highest stiffness. It has a lower weight-to-torque ratio than all springs except spring K. Despite being slightly heavier than springs L, K, E, and D, it can still be considered light compared to springs A, C, and F. The maximum angular deflection was higher than springs L and D but lower than springs A, C, F, and K due to its high stiffness. The torque resolution is proportional to the stiffness, and the maximum angular deflection is inversely proportional to the stiffness. Therefore, the proposed spring has a higher torque resolution than springs A, C, F, and K and a lower torque resolution than springs L and D.

Regarding compactness, the proposed spring is smaller than springs L, K, E, D, and B but larger than springs A, C, and F. The compactness of a spring can be measured by the volume of the spring, calculated by multiplying its cross-sectional area (diameter) by its length. 

It is important to note that spring F has a higher maximum angular deflection than the proposed spring, but the proposed spring has better weight, weight-to-torque ratio, and compactness characteristics. 

**Figure 15:**
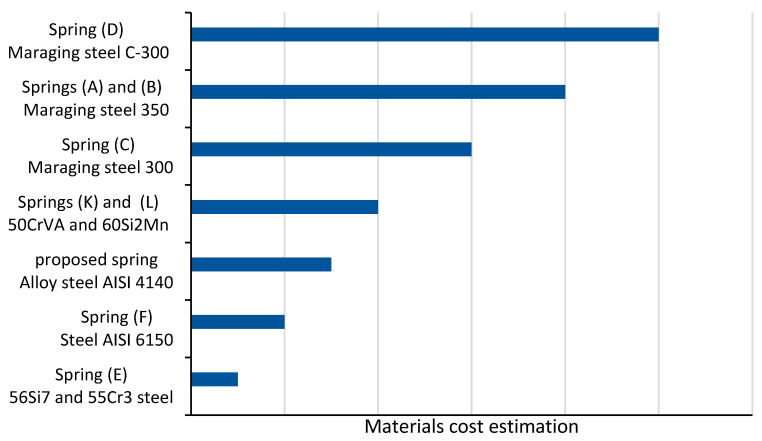
Materials cost estimation for torsion springs. (A) [15]. (B) [10]. (C) [16]. (D) [7]. (E) [17]. (F) [18]. (K) [25]. (L) [22].

The cost of the materials used in the proposed spring, chromium-molybdenum alloy steel AISI 4140, was estimated to be more cost-effective than other materials used in the literature except for springs E and F in Figure 15. Spring E does not have better characteristics overall compared to the proposed spring. The proposed spring has better characteristics than spring F in terms of weight and weight-to-torque ratio and similar characteristics in terms of compactness. The only characteristic where spring F exceeds the proposed spring is maximum angular deflection. 

Overall, the proposed torsion spring has excellent characteristics, including the highest stiffness and a lower weight-to-torque ratio, and its material cost is more cost-effective than other springs. 

## 6. Conclusions and Recommendations

In conclusion, the torsion spring module presented in this study provides a cost-effective and efficient solution for the lumbar support exoskeleton of the ES-RSEA. The prototype of the torsion spring was designed, modelled, and evaluated based on its spring and stiffness characteristics, incorporating DFM principles for lightweight, compact, cost-efficient, and easy-to-manufacture design. The FEM-based design optimization was validated by comparing the simulation and experimental results, which showed good agreement. The torsion spring demonstrated a maximum torque capacity of 45.7 Nm and a stiffness of 440 Nm/rad, consistent with the FEM simulations with a deviation of roughly 1.166%. The stiffness characteristics were linear, with the minimal deviation between measurements and simulations. The chosen material, AISI 4140 alloy steel, was cost-effective compared to other materials in the literature. The proposed torsion spring has a good balance between stiffness, weight-to-torque ratio, and compactness, making it a promising candidate for use in the lumbar support exoskeleton. Overall, the study demonstrates the excellent characteristics of the proposed torsion spring, including low stiffness, low weight-to-torque ratio, and cost-effectiveness compared to other springs in the literature.

Based on the results of this study, it is recommended that the proposed torsion spring design be considered for applications that require low stiffness with better characteristics, low weight-to-torque ratio, and cost-effectiveness. Additionally, further research can be conducted to modify the parametric CAD modelling design for use in different SEA applications, such as pHRI and humanoid or walking robots. The DFM-based design methodology used in this study can also be applied to other elastic elements in compliance actuators with appropriate modifications. It is also suggested that consideration is given to trimming the unmounted part of the outer ring to reduce the overall mass by up to 20% as a potential enhancement.

## Figures and Tables

**Table 2:** Design requirements of the torsional spring.

Design Parameter	Desired Value for the Proposed Design
Output torque of the RSEA	45.7 Nm
Maximum thickness	10 mm
Maximum outer diameter	85 mm
Minimum inner diameter	20 mm
Torsional spring stiffness	200–800 Nm/rad
Maximum weight	150 g
Spring material	Low-alloy steel AISI 4140
yield strength	1400 MPA
Angular deflection	5.8 Degree
Weight-to-torque ratio	3 g/Nm

**Table 3:** Search space parameters for the chosen topology.

Parameter	Minimum	Maximum	Minimum Increment
R3	25	33	0.2
R4	22	32	0.2
R5	23	33	0.5
T2	1	4	0.1
T3	3	7	0.1
T4	4.5	16	0.5
t	4	16	2

**Table 4:** Number of nodes produced for finite elements design.

Mesh Property/Spring Type	Number of Nodes Produced
Element Quantity Fluctuation	100,000 to 500,000
Convergence	250,000–300,000
Actual optimization	292,842

**Table 5:** Optimized parameters for the chosen topology.

Parameter	Optimized
R3	32.8
R4	31.2
R5	28.5
T2	1.6
T3	5.2
T4	8.5
T	8

## Data Availability

All the relevant data are within the paper.
